# Association between dietary vitamin K intake and lipid metabolism among populations with cardiovascular disease

**DOI:** 10.3389/fnut.2025.1605300

**Published:** 2025-07-04

**Authors:** Tao Liu, Lili Wang, Qiming Dai, Yesheng Pan

**Affiliations:** ^1^Department of Cardiology, Zhongda Hospital, School of Medicine, Southeast University, Nanjing, China; ^2^Department of Cardiology, The Second Affiliated Hospital of Xuzhou Medical University, Xuzhou, China; ^3^Department of Cardiology, Jinshan Branch of Shanghai Sixth People’s Hospital, Shanghai, China

**Keywords:** dietary vitamin K, intake, lipid metabolism, cardiovascular disease, NHANES

## Abstract

**Background:**

The objective of this investigation was to examine the correlation between intake of dietary vitamin K and lipid metabolism in cardiovascular disease populations.

**Methods:**

The data for this investigation were obtained from the National Health and Nutrition Examination Survey (NHANES) 2003–2014. The exposure variable was the total daily intake of dietary vitamin K (μg). Triglycerides (TG), total cholesterol (TC), high-density lipoprotein cholesterol (HDL-C), and low-density lipoprotein cholesterol (LDL-C) comprised the lipid indicators. To investigate the relationship between vitamin K intake and lipid metabolism, the following analyses were conducted: weighted multiple linear regression, smoothing curve fitting, generalized additive models, threshold analysis, subgroup analysis, and sensitivity analyses.

**Results:**

Ultimately, 1,543 participants aged 18 years or older were enrolled. The total daily intake of dietary vitamin K was found to be negatively correlated with TG (β: −15.57, 95% CI: −27.806, −3.333) and TC (β: −6.564, 95% CI: −12.252, −0.877). For each 1 ug increase in the total daily intake of dietary vitamin K, the LDL-C would decrease by 0.510 mg/dl (95% CI: −0.940, −0.078) when the total daily intake of dietary vitamin K was less than 23.7 ug. HDL-C was not influenced by total daily intake of dietary vitamin K. Furthermore, subgroup analyses and sensitivity analyses revealed that an increase in the total daily intake of dietary vitamin K was still negatively associated with TG, TC, and LDL.

**Conclusion:**

The consumption of foods with high vitamin K levels might contribute to the improvement of TC, TG, and LDL-C levels in CVD populations.

## Introduction

Cardiovascular disease (CVD) remains a substantial cause of mortality on a global scale (1), and it is influenced by a variety of risk factors, including hypertension, chronic inflammation, and dyslipidemia (2, 3). Lipid metabolism has attracted significant attention due to its critical role in the development of atherosclerosis, the primary cause of CVD (4, 5). Additionally, dysregulated lipid profiles, which include increased low-density lipoprotein cholesterol (LDL-C), total cholesterol (TC) and triglycerides (TG) and reduced high-density lipoprotein cholesterol (HDL-C), can affect the formation of plaque, endothelial dysfunction, and vascular complications in patients with CVD, potentially exacerbating the severity of it. For this reason, the treatment of lipid disorders may be the most critical therapeutic approach to reducing the prevalence of CVD (6).

Recent research has underscored the significance of dietary factors in the regulation of lipid metabolism, with a particular emphasis on micronutrients such as vitamins (7–10). Vitamin K is a fat-soluble vitamin among these. Previous research has demonstrated its critical role in the condition of bones, calcium metabolism, and blood coagulation (11, 12). However, Lider (13) discovered that the administration of additional vitamins K and E to rodents undergoing adrenalectomy prevented these changes in membrane phospholipid composition. Consequently, a potential effect of vitamin K intake on lipid metabolism is possible. Nevertheless, there are a limited number of studies that investigate the impact of daily intake of dietary vitamin K on lipid profiles, particularly in populations with CVD.

Understanding the influence of dietary vitamin K intake on lipid metabolism in populations with CVD is of paramount importance, given the influence of lipid levels on CVD. Consequently, we posited that an increase in the ingestion of dietary vitamin K would be linked to an improvement in the lipid metabolism levels of CVD populations. We conducted a cross-sectional analysis of data from the National Health and Nutrition Examination Survey (NHANES) in the present study to assess the correlation between dietary vitamin K intake and lipid metabolism in populations with CVD. We hoped that the findings of this study could provide a reference for nutritional intervention to improve the prognosis of CVD populations.

## Materials and methods

### Study population

**FIGURE 1:**
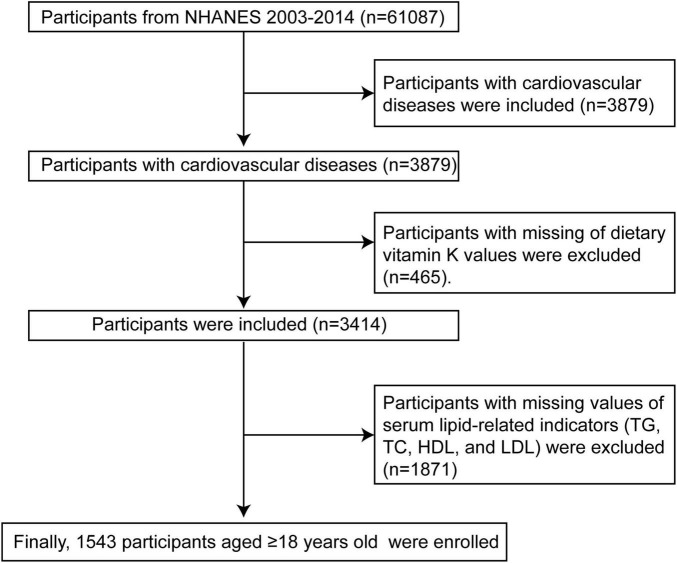
Flow chart of CVD participants selection from the NHANES 2003–2014. TG, triglycerides; TC, total cholesterol; HDL-C, high-density lipoprotein cholesterol; LDL-C, low-density lipoprotein cholesterol; NHANES, National Health and Nutrition Examination Survey.

National Health and Nutrition Examination Survey was a cross-sectional survey that was intended to evaluate the health and nutritional status of adults and children in the United States. In order to guarantee that the non-institutionalized United States population is adequately represented, NHANES implements a multistage probability sampling design that is intricate. Through interviews and physical examinations, including laboratory measurements, the survey gathers demographic, dietary, and health-related data. The data for the present investigation were obtained from the NHANES 2003–2014. A total of 61,087 participants were incorporated into these six cycles. As illustrated in Figure 1, 57,208 of the 61,087 participants were excluded due to no CVD or absence of CVD records. Out of the 3,879 remaining CVD participants, 465 were excluded due to incomplete dietary vitamin K intake values. Finally, 1,543 populations aged 18 years or older were enrolled after 1,871 participants were excluded due to lacking values for lipid-related indicators. Based on data acquired from personal interviews, CVD was defined as the self-reported history of coronary heart disease (CHD), angina, myocardial infarction (MI), congestive heart failure (CHF), and stroke (14).

### Dietary vitamin K intake

The purpose of the dietary interview component is to obtain detailed dietary intake information from NHANES participants. Dietary intake data are used to estimate the types and amounts of food and beverages (including all types of water) consumed in the 24 h before the interview (midnight to midnight) and to estimate the energy, nutrients, and other food components consumed from these foods and beverages. The dietary interview component, called What We Eat in America (WWEIA), is a collaborative effort between the United States Department of Agriculture (USDA) and the United States Department of Health and Human Services (DHHS). Under this collaboration, the National Center for Health Statistics (NCHS) of DHHS is responsible for all aspects of survey sample design and data collection, while the USDA Food Survey Research Group (FSRG) is responsible for dietary data collection methods, maintenance of the database used to code and process data, and data review and processing. Recall interviews will be collected on-site at mobile examination centers (MECs). The exposure variable in this study is total dietary vitamin K intake (μg) recorded on the first day in NHANES 2003–2014, including dietary sources and supplements. Data are obtained during a 24-h dietary recall interview with participants at MECs.

### Measurement of lipid profiles

Blood samples were collected in accordance with a standardized protocol that was delineated on the NHANES website by trained personnel. The NHANES Laboratory Operations Manual^1^ contains comprehensive sample collection procedures. TG, TC, HDL-C, and LDL-C were the primary lipid indicators examined in this study.

### Covariates

The following covariates were chosen based on the relationship between covariates and lipid profiles, cardiovascular disease, or vitamin intake, as per previous publications (15–19). Covariates included in the present study contained sociodemographic data, laboratory data, diet, physical examination, life history, and comorbidity. Continuous variables were as follows: age (years), poverty-income ratio (PIR), and body mass index (BMI, kg/m^2^). Categorical variables were as follows: sex (Male/Female), ethnicity (White/Mexican/Black/Other), education level (Less than high school graduate/High school graduate or general equivalency diploma/Some college or above), marital status (Married/Other), smoking status (Never/Former/Now), drinking status (Never/Former/Now), diabetes mellitus (DM, No/Yes), hypertension (No/Yes), chronic kidney disease (CKD, No/Yes), and lipid-lowering drugs (No/Yes).

### Statistical analysis

The complex sampling design principles employed in NHANES (weights = 1/6 * WTMEC2YR) were adhered to in this investigation. Furthermore, in order to resolve the issue of missing values in the covariates, multiple imputation was implemented. Previous research has demonstrated that estimates derived through multiple imputation methods may be biased when the number of absent values for variables exceeds 10% (20). The maximum percentage of missing values among all variables in this analysis was 8.23% (Supplementary Table 1). Consequently, the present investigation deemed the use of multiple imputation to be appropriate. The clinical characteristics at baseline are organized by the tertiles of total daily dietary vitamin K intake. The three tertiles are as follows: tertile 1 (0–35.7 ug), tertile 2 (35.7–75.4 ug), and tertile 3 (75.4–1285.4 ug). The mean ± standard deviation (SD) was used to express variables that followed a normal distribution, and the *t*-test was employed to compare differences between groups. The variables that did not follow a normal distribution were expressed as median and interquartile range (IQR), and the Wilcoxon rank-sum test or Kruskal–Wallis test was employed to compare differences between groups. The categorical variables were expressed as percentages and integers, and χ^2^ test was employed to compare the differences between groups.

The specific association between total daily intake of dietary vitamin K and lipid-related parameters in the CVD populations was determined by the construction of multiple linear regression models in this study. No variable was unadjusted in model 1. Age, sex, ethnicity, marital status, poverty income ratio, BMI, and education level were adjusted in Model 2. Age, sex, ethnicity, marital status, poverty income ratio, BMI, education level, smoking status, alcohol status, diabetes mellitus, hypertension, chronic kidney disease, and treatment with lipid-lowering medications were adjusted in Model 3. The curvilinear association between total daily intake of dietary vitamin K and lipid-related parameters was evaluated using generalized additive models and smoothing curve fitting. Threshold effects were employed to determine the inflection point of change in the relationship between total daily intake of dietary vitamin K and lipid-related parameters when non-linear relationships were identified. Furthermore, we conducted interaction and subgroup analyses based on age (≤ 65 years/> 65 years), sex (male/female), BMI (≤ 30 kg/m^2^/> 30 kg/m^2^), DM (Yes/No), hypertension (Yes/No), and CKD (Yes/No). We also conducted the threshold analysis of subgroups for the variable (DM) that interacted with dietary vitamin K.

In order to evaluate the stability of the results, we conducted sensitivity analyses. Specifically, we conducted multiple linear regression, generalized additive models, and threshold analyses to investigate the relationship between dietary vitamin K intake and lipid-related parameters after removing all missing values of all covariates. *P*-value < 0.05 indicated that the difference was statistically significant. All statistical analysis was performed using R 4.4.0 software.

## Results

Table 1 displays the clinical characteristics of the research population at baseline. The present study included a total of 1,543 participants aged 18 years or older, with a mean (SD) age of 66.95 (12.87) years. The participants were divided between 877 (56.84%) males and 666 (43.16%) females. In comparison to the first tertile of total daily intake of dietary vitamin K, the population in the third tertile of total daily intake of dietary vitamin K was more likely to be male (64.41% vs. 46.86%; *P*-value < 0.001) and White (61.90% vs. 57.06%; *P*-value < 0.01), have some college or above (46.62% vs. 31.37%; *P*-value < 0.001), be married (59.19% vs. 47.84%; *P*-value < 0.01), be not smokers (16.44% vs. 27.06%; *P*-value < 0.001), be now drinkers (54.16% vs. 42.16%; *P*-value < 0.001), and have higher levels of PIR (median [IRQ], 2.29[2.78] vs. 1.49[1.53]; *P*-value < 0.001), lower levels of TG (median [IRQ], 112.00[86.00] vs. 129.00[102.00]; *P*-value < 0.001), lower levels of TC (median [IRQ], 174.00[58.00] vs. 185.00[53.00]; *P*-value < 0.001) and lower levels of LDL-C (median [IRQ], 101.50[51.75] vs. 96.00[46.00]; *P*-value = 0.02). There were no significant differences in HDL-C, age, BMI, DM, hypertension, CKD and lipid-lowering drugs between the two groups.

**TABLE 1:** Baseline characteristics of 1,543 participants according to tertiles of vitamin K.

Variable	Total (*n* = 1,543)	Tertiles of vitamin K, ug			*P*-value
		T1 (*n* = 510)	T2 (*n* = 516)	T3 (*n* = 517)	
Age, mean (SD), years	66.95 (12.87)	66.38 (13.52)	67.91 (11.87)	66.56 (13.12)	0.12
**Sex, *n* (%)**					< 0.001
Male	877 (56.84)	239 (46.86)	305 (59.11)	333 (64.41)	
Female	666 (43.16)	271 (53.14)	211 (40.89)	184 (35.59)	
**Ethnicity, *n* (%)**					< 0.01
White	915 (59.30)	291 (57.06)	304 (58.91)	320 (61.90)	
Mexican	157 (10.17)	60 (11.76)	68 (13.18)	29 (5.61)	
Black	308 (19.96)	106 (20.78)	90 (17.44)	112 (21.66)	
Other	163 (10.56)	53 (10.39)	54 (10.47)	56 (10.83)	
**Education level, *n* (%)**					< 0.001
Less than high school graduate	563 (36.49)	231 (45.29)	182 (35.27)	150 (29.01)	
High school graduate or general equivalency diploma	376 (24.37)	119 (23.33)	131 (25.39)	126 (24.37)	
Some college or above	604 (39.14)	160 (31.37)	203 (39.34)	241 (46.62)	
**Marital status, *n* (%)**					< 0.01
Married	832 (53.92)	244 (47.84)	282 (54.65)	306 (59.19)	
Other	711 (46.08)	266 (52.16)	234 (45.35)	211 (40.81)	
PIR, median (IRQ)	1.83 (2.11)	1.49 (1.53)	1.83 (1.43)	2.29 (2.78)	< 0.001
BMI, mean (SD), kg/m^2^	30.14 (6.95)	30.24 (7.36)	30.07 (6.63)	30.10 (6.85)	0.91
**Smoking status, *n* (%)**					< 0.001
Never	611 (39.60)	210 (41.18)	197 (38.18)	204 (39.46)	
Former	609 (39.47)	162 (31.76)	219 (42.44)	228 (44.10)	
Now	323 (20.93)	138 (27.06)	100 (19.38)	85 (16.44)	
**Drinking status, *n* (%)**					< 0.001
Never	219 (14.19)	94 (18.43)	65 (12.60)	60 (11.61)	
Former	573 (37.14)	201 (39.41)	195 (37.79)	177 (34.24)	
Now	751 (48.67)	215 (42.16)	256 (49.61)	280 (54.16)	
**DM, *n* (%)**					0.95
No	882 (57.16)	289 (56.67)	295 (57.17)	298 (57.64)	
Yes	661 (42.84)	221 (43.33)	221 (42.83)	219 (42.36)	
**Hypertension, *n* (%)**					0.21
No	340 (22.03)	126 (24.71)	107 (20.74)	107 (20.70)	
Yes	1,203 (77.97)	384 (75.29)	409 (79.26)	410 (79.30)	
**CKD, *n* (%)**					0.34
No	824 (53.40)	272 (53.33)	264 (51.16)	288 (55.71)	
Yes	719 (46.60)	238 (46.67)	252 (48.84)	229 (44.29)	
**Lipid-lowering drugs, *n* (%)**					0.84
No	678 (43.94)	227 (44.51)	241 (44.54)	210 (42.68)	
Yes	865 (56.06)	283 (55.49)	300 (55.45)	282 (57.32)	
**Serum lipid-related indicators, median (IRQ), mg/dl**					
Triglycerides	121.00 (91.00)	129.00 (102.00)	123.50 (90.5)	112.00 (86.00)	< 0.001
Total cholesterol	177.00 (57.00)	185.00 (53.00)	176.00 (59.25)	174.00 (58.00)	< 0.001
HDL cholesterol	48.00 (18.00)	49.00 (19.00)	48.00 (18.00)	48.00 (18.00)	0.76
LDL cholesterol	98.00 (49.00)	101.50 (51.75)	97.50 (50.00)	96.00 (46.00)	0.02

T1: 0–35.7 ug; T2: 35.7–75.4 ug; T3: 75.4–1,285.4 ug. HDL, high-density lipoprotein; LDL, low- density lipoprotein; PIR; poverty income ratio; BMI, body mass index; DM, diabetes mellitus; CKD, chronic kidney disease; IQR, interquartile range (75th quartile minus 25th quartile).

**FIGURE 2:**
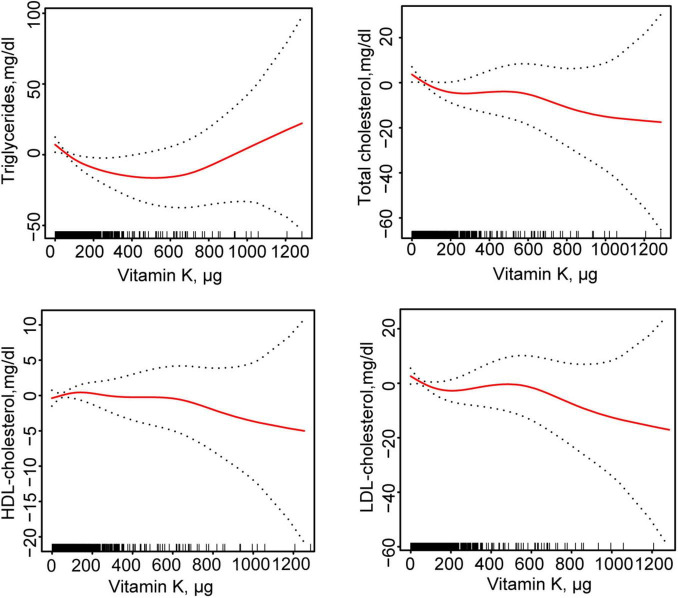
Fitting curves of association between dietary vitamin K intake and lipid-related indicators among CVD populations. Age, sex, ethnicity, marital status, poverty income ratio, BMI, education level, smoking status, drinking status, DM, hypertension, CKD, and lipid-lowering drugs were adjusted. HDL, high-density lipoprotein; LDL, low- density lipoprotein; BMI, body mass index; DM, diabetes mellitus; CKD, chronic kidney disease; CVD, cardiovascular disease.

Table 2 showed the association between total daily intake of dietary vitamin K and lipid-related parameters in models 1, 2, and 3. After full adjustment for potential confounders (Model 3), compared with the first tertile of total daily intake of dietary vitamin K, for every 1 ug increase in the third tertile of total daily intake of dietary vitamin K, the changes in β (95% CI) values for TG and TC were −15.57 (−27.806, −3.333) mg/dl and −6.564 (−12.252, −0.877) mg/dl, respectively. Using generalized additive models and smooth curve fitting, we found that there was a non-linear association between total daily intake of dietary vitamin K and LDL (Figure 2).

**TABLE 2:** Association between dietary vitamin K intake and lipid-related indicators among CVD populations.

Outcomes	Model 1	Model 2	Model 3
	β 95% CI	β 95% CI	β 95% CI
**Triglycerides**			
Vitamin K	−0.042 (−0.082, −0.001)*	−0.039 (−0.075, −0.004)*	−0.038 (− 0.073, −0.003)*
**Tertiles of vitamin K**			
T1	Ref	Ref	Ref
T2	−5.965 (−15.690, 3.760)	−5.275 (−13.876, 3.326)	−4.86 (−13.585, 3.865)
T3	−15.129 (−29.356, −0.902)*	−15.885 (−28.193, −3.578)*	−15.57 (−27.806, −3.333)*
**Total cholesterol**			
Vitamin K	−0.015 (−0.047, 0.017)	−0.005 (−0.034, 0.025)	−0.006 (−0.035, 0.024)
**Tertiles of vitamin K**			
T1	Ref	Ref	Ref
T2	−5.604 (−12.419, 1.211)	−1.938 (−8.581, 4.705)	−2.785 (−9.131, 3.560)
T3	−11.021 (−17.025, −5.017)***	−5.223 (−10.787, 0.342)	−6.564 (−12.252, −0.877)*
**HDL cholesterol**			
Vitamin K	−0.001 (−0.009, 0.006)	0.002 (−0.004, 0.007)	0.001 (−0.004, 0.006)
**Tertiles of vitamin K**			
T1	Ref	Ref	Ref
T2	0.043 (−2.214, 2.300)	1.074 (−1.033, 3.180)	0.79 (−1.164, 2.744)
T3	−0.47 (−3.107, 2.167)	1.585 (−0.703, 3.874)	1.237 (−0.924, 3.399)
**LDL cholesterol**			
Vitamin K	−0.006 (−0.034, 0.022)	0.002 (−0.024, 0.027)	0.001 (−0.024, 0.026)
**Tertiles of vitamin K**			
T1	Ref	Ref	Ref
T2	−4.472 (−10.391, 1.447)	−1.971 (−7.691, 3.750)	−2.619 (−8.182, 2.944)
T3	−7.524 (−12.401, −2.647)**	−3.628 (−8.224, 0.968)	−4.685 (−9.437, 0.068)

*P-value < 0.05; **P-value < 0.01; ***P-value < 0.001. T1: 0–35.7 ug; T2: 35.7–75.4 ug; T3: 75.4–1,285.4 ug, Model 1 did not adjust any variables. Model 2 adjusted age, sex, ethnicity, marital status, poverty income ratio, BMI, and education level. Model 3 adjusted age, sex, ethnicity, marital status, poverty income ratio, BMI, education level, smoking status, drinking status, DM, hypertension, CKD, and lipid-lowering drugs. HDL, high-density lipoprotein; LDL, low-density lipoprotein; BMI, body mass index; DM, diabetes mellitus; CKD, chronic kidney disease; CVD, cardiovascular disease.

According to the segmented linear regression, the inflection points of dietary vitamin K intake in relation to TG, TC, and LDL were 123.7, 24.1, and 23.7 ug, respectively, after thoroughly accounting for confounding factors. The log-likelihood ratio test verified the significance of inflection points (*P*-value < 0.05). Initially, TG would decrease by 0.156 mg/dl (95% CI: −0.254, −0.057) for every 1 ug increase in total daily intake of dietary vitamin K in the general population for TG below the inflection point (123.7 ug). However, there was no statistical significance between total daily intake of dietary vitamin K and TG after this point. Secondly, for TC, below the inflection point (24.1 ug), a 0.618 mg/dl decrease in TC levels would result for every 1 ug increase in total daily intake of dietary vitamin K in the general population (95% CI: −1.107, −0.129). However, there was no statistical significance between total daily intake of dietary vitamin K and TC after this point. Lastly, for LDL-C, below the inflection point (23.7 ug), a 0.510 mg/dl decrease in LDL-C would result for every 1 ug increase in total daily intake of dietary vitamin K in the general population (95% CI: −0.940, −0.078). After this point, there was no statistically significant correlation between LDL-C and total daily intake of dietary vitamin K (Table 3).

**TABLE 3:** Threshold effect analysis of dietary vitamin K intake on lipid-related indicators.

Outcomes	β (95% CI)	*P*-value
**Triglyceride**		
Inflection point	123.7 ug	
≤ 123.7 ug	−0.156 (−0.254, −0.057)	0.002
> 123.7 ug	−0.006 (−0.043, 0.032)	0.767
Log-likelihood ratio	0.013	
**Total cholesterol**		
Inflection point	24.1 ug	
≤ 24.1 ug	−0.618 (−1.107, −0.129)	0.013
> 24.1 ug	−0.015 (−0.033, 0.004)	0.118
Log-likelihood ratio	0.016	
**HDL cholesterol**		
Inflection point	10.5 ug	
≤ 10.5 ug	−0.611 (−1.283, 0.061)	0.075
> 10.5 ug	0.001 (−0.005, 0.007)	0.845
Log-likelihood ratio	0.073	
**LDL cholesterol**		
Inflection point	23.7 ug	
≤ 23.7 ug	−0.510 (−0.940, −0.078)	0.021
> 23.7 ug	−0.008 (−0.024, 0.007)	0.302
Log-likelihood ratio	0.023	

Age, sex, ethnicity, marital status, poverty income ratio, BMI, education level, smoking status, drinking status, DM, hypertension, CKD, and lipid-lowering drugs were adjusted. HDL, high-density lipoprotein; LDL, low-density lipoprotein; BMI, body mass index; DM, diabetes mellitus; CKD, chronic kidney disease.

**FIGURE 3:**
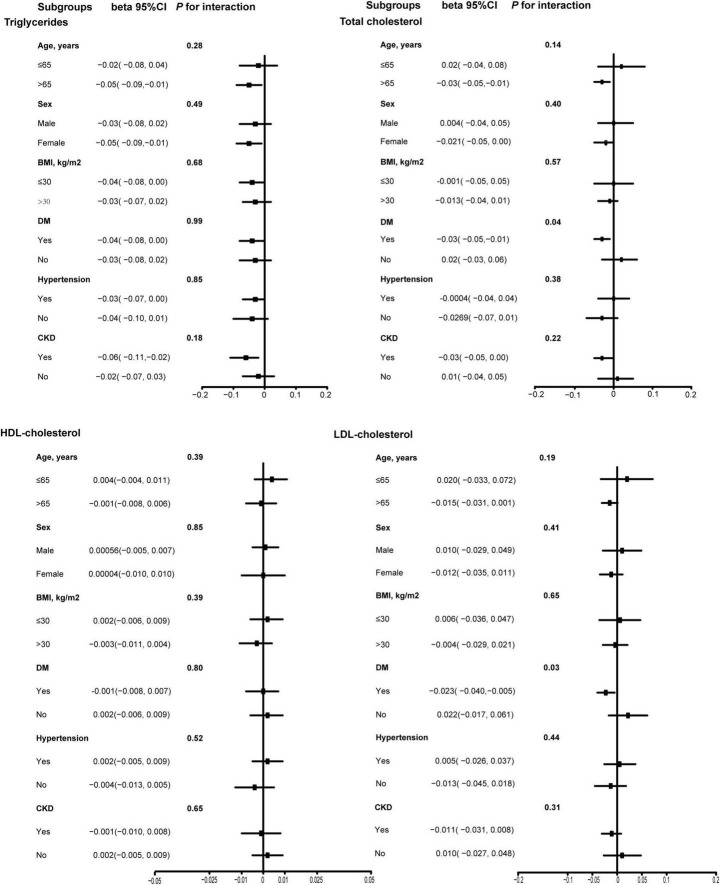
Subgroup analysis of association between dietary vitamin K intake and lipid-related indicators among CVD populations. Age, sex, ethnicity, marital status, poverty income ratio, BMI, education level, smoking status, drinking status, DM, hypertension, CKD, and lipid-lowering drugs were adjusted. HDL, high-density lipoprotein; LDL, low- density lipoprotein; BMI, body mass index; DM, diabetes mellitus; CKD, chronic kidney disease; CVD, cardiovascular disease.

We performed subgroup analyses based on age (≤ 65 years/> 65 years), sex (male/female), BMI (≤ 30 kg/m^2^/> 30 kg/m^2^), DM (Yes/No), hypertension (Yes/No), and CKD (Yes/No). The results in Figure 3 showed that there was a significant interaction for DM on association of total daily intake of dietary vitamin K with TC and LDL-C in these subgroups. Therefore, for effect total daily intake of dietary vitamin K on TC and LDL-C levels in the DM subgroup, we also performed generalized additive models and smooth curve fitting, and found that there was a non-linear association between total daily intake of dietary vitamin K and TC and LDL-C among CVD populations without DM (Supplementary Figure 1). Therefore, we performed the threshold effect analysis among CVD populations without DM, and found total daily intake of dietary vitamin K was negatively associated with TC and LDL-C without DM when a certain amount of dietary vitamin K was consumed (Supplementary Table 2).

To assess the stability of the results, after eliminating all missing values of covariates, we performed again multiple linear regression, generalized additive models, and threshold analyses among the 1,297 CVD populations to explore the association between total daily intake of dietary vitamin K and lipid-related parameters, found that increase of total daily intake of dietary vitamin K was still associated with improvements of TG levels, TC levels, and LDL levels (Supplementary Tables 3, 4 and Supplementary Figure 2).

## Discussion

The present study examined the relationship between the total daily intake of dietary vitamin K and the levels of TG, TC, HDL-C, and LDL-C in CVD populations. The total daily intake of dietary vitamin K might be found to be beneficial in the improvement of TG (β: −15.57, 95% CI: −27.806, −3.333) and TC (β: −6.564, 95% CI: −12.252, −0.877). Furthermore, the LDL-C level would decrease by 0.510 mg/dl (95% CI: −0.940, −0.078) for every 1 ug increase in total daily intake of dietary vitamin K when the total daily intake of dietary vitamin K was less than 23.7 ug. Nevertheless, the total daily intake of dietary vitamin K did not have any impact on HDL-C levels in CVD populations. These findings might offer a reference for the use of vitamin K intake as a dietary intervention in the treatment of dyslipidemia.

Previous studies have demonstrated that an increase in the total daily intake of dietary vitamin K could significantly enhance the mass of skeletal muscle in men (β = 0.05747, *p* = 0.0204) (21). It was discovered by Wang et al. (22) that the risk of depression might be reduced by an increase in Vitamin K intake, as it mediated oxidative homeostasis. Wu et al. (23) reported that the relationship between atherosclerotic cardiovascular disease (ASCVD) and the dietary vitamin K was L-shaped (non-linear, *p* = 0.006). The odds ratio (OR) for ASCVD was 0.996 (95% CI: 0.993–0.998, *P* = 0.002) for participants with a vitamin K intake of less than 127.1 ug/day (23). The research findings cited above demonstrated that the health of humans was improved by increasing the ingestion of vitamin K.

Additionally, one study in rats showed that long-term supplementation of phylloquinone or MK-4 significantly reduced total fat accumulation (24). Varsamis et al. (25) found that increased dietary intake of vitamin K2 was associated with reduced LDL-C levels. This means that an increase in the total daily intake of dietary vitamin K may effectively improve blood lipid levels. Currently, the relationship between intake of vitamin K and lipid metabolism among CVD populations is still unclear. This study might be the first to report the effect of dietary vitamin intake on lipid levels in CVD populations, and found an increase in the total daily intake of dietary vitamin K might be beneficial to improvement of TG levels, TC levels, and LDL-C levels among CVD populations. Additionally, there was a significant interaction for DM on association of total daily intake of dietary vitamin K with TC (*P* for interaction = 0.04) and LDL-C (*P* for interaction = 0.03) in subgroup analysis. Vitamin K intake can reduce the risk of type 2 DM. And it helps improve glucose control and insulin resistance in patients with type 2 DM (26, 27). In addition, insulin resistance may reduce the efficiency of vitamins in cells, thereby affecting their ability to regulate lipid metabolism. In contrast, in individuals without DM, vitamins may be more effectively involved in lipid metabolism regulation (28, 29). This may explain why diabetes affected the association between vitamin K and lipid levels in the present study. Moreover, systemic inflammation and oxidative stress in diabetes could attenuate vitamin K for anti-inflammatory and antioxidant effects on LDL oxidation. For instance, hyperglycemia-induced advanced glycation end products (AGEs) may interfere with vitamin K’s ability to inhibit pro-inflammatory cytokines like TNF-α (29). These mechanisms collectively suggest that diabetes alters the bioavailability or cellular responsiveness to vitamin K, though further experimental studies are warranted to validate these pathways.

The current prevention and treatment guidelines for CVD underscore the significance of dietary modification, particularly in relation to cholesterol and lipid intake (30–34). Due to its potential clinical significance, intake of a certain amount of vitamin K may serve as an important means of dietary intervention to improve lipid metabolism. Based on the results of this study, intake of a certain dose of dietary vitamin K might be effective in improving lipid profiles in the CVD populations. The possible mechanisms by which vitamin K improves lipids levels include the following: Firstly, vitamin K activates Gla-dependent proteins in the liver, which helps in lipid metabolism and regulation (35). Secondly, vitamin K could inhibit the production of pro-inflammatory cytokines such as IL-6 and TNF-α. It reduces lipid deposition by reducing damage to the vascular lining, thereby improving blood lipid metabolism (36, 37). Finally, vitamin K is believed to be able to reduce the generation of free radicals through its antioxidant properties, protect LDL-C from oxidation, and prevent the further development of atherosclerosis (38, 39).

This study has several key strengths, including its analysis of a large, nationally representative NHANES cohort (2003–2014; *n* = 1,543 CVD patients), ensuring robust statistical power and generalizability. The research employed rigorous statistical adjustments for sociodemographic, metabolic, and lifestyle confounders, consistently demonstrating that higher dietary vitamin K intake was associated with lower TG, TC, and LDL-C levels. Notably, it identified non-linear threshold effects, revealing that LDL-C significantly decreased only when vitamin K intake was below 23.7 μg/day-a novel finding with potential clinical implications for dietary interventions. Additionally, subgroup and sensitivity analyses reinforced the reliability of the results. Although our investigation offers several insights, it is imperative to recognize several constraints. To begin, the cross-sectional design of NHANES restricts our capacity to establish causality between lipid metabolism and the total daily intake of dietary vitamin K. In order to verify these associations and ascertain whether the consumption of vitamin K can result in long-term enhancements in lipid profiles, longitudinal studies should be required. Additionally, the 24-h recall method was employed to evaluate dietary consumption, which may not accurately reflect long-term intake and may be subject to recall bias. Notably, sensitivity analyses suggested the robustness of this study. The future studies should use more accurate dietary assessment methods, such as diet records. Finally, Vitamin K from different food sources may have different impacts on lipid metabolism. For example, vitamin K1 from green leafy vegetables and vitamin K2 from animal liver may differ in their physiological effects. The future studies conduct a classification analysis of the sources of vitamin K intake to more accurately assess its impact on lipid metabolism.

In conclusion, our study found that intake of dietary vitamin K might improve lipid levels in the American CVD populations to a certain extent, which might provide a certain reference for further research on the improvement of CVD prognosis by a balanced diet.

## Conclusion

This study revealed that there might be a relationship between daily intake of dietary vitamin K and lipid metabolism in the CVD population. TC, TG, and LDL-C might be regulated by the daily intake of dietary vitamin K. Increasing daily intake of dietary vitamin K was beneficial to improve TG and TC. In addition, when daily intake of dietary vitamin K was less than 23.7 ug, LDL-C reduced as vitamin K intake increased.

## Data Availability

Publicly available datasets were analyzed in this study. This data can be found here: https://www.cdc.gov/nchs/nhanes.

## Footnotes

1
https://www.cdc.gov/nchs/nhanes/index.htm
