# Supplementary material for: Association between dietary vitamin K intake and lipid metabolism among populations with cardiovascular disease
Source: Front Nutr. 2025 Jul 4;12:1605300. doi: 10.3389/fnut.2025.1605300 (PMC12270866; doi:10.3389/fnut.2025.1605300)
Supplement: Supplementary file 1 [file Data_Sheet_1.docx]

**Table S1.** Missing values ​​in this study

| Variables | Numbers of missing values (N) | Missing proportion (%) | Total (N) |
| --- | --- | --- | --- |
| Age | 0 | 0 | 1543 |
| Sex | 0 | 0 | 1543 |
| Ethnicity | 0 | 0 | 1543 |
| Marital status | 1 | 0.06 | 1543 |
| Poverty income ratio | 127 | 8.23 | 1543 |
| Education level | 6 | 0.39 | 1543 |
| Smoking status | 1 | 0.06 | 1543 |
| Drinking status | 85 | 5.51 | 1543 |
| Body mass index | 40 | 2.59 | 1543 |
| Diabetes mellitus | 2 | 0.13 | 1543 |
| Hyperlipidemia | 0 | 0 | 1543 |
| Hypertension | 0 | 0 | 1543 |
| Chronic kidney disease | 24 | 1.56 | 1543 |
| Lipid-lowering drugs | 0 | 0 | 1543 |
| Vitamin K | 0 | 0 | 1543 |
| Triglycerides | 0 | 0 | 1543 |
| Total cholesterol | 0 | 0 | 1543 |
| Hdl-cholesterol | 0 | 0 | 1543 |
| LDL-cholesterol | 0 | 0 | 1543 |


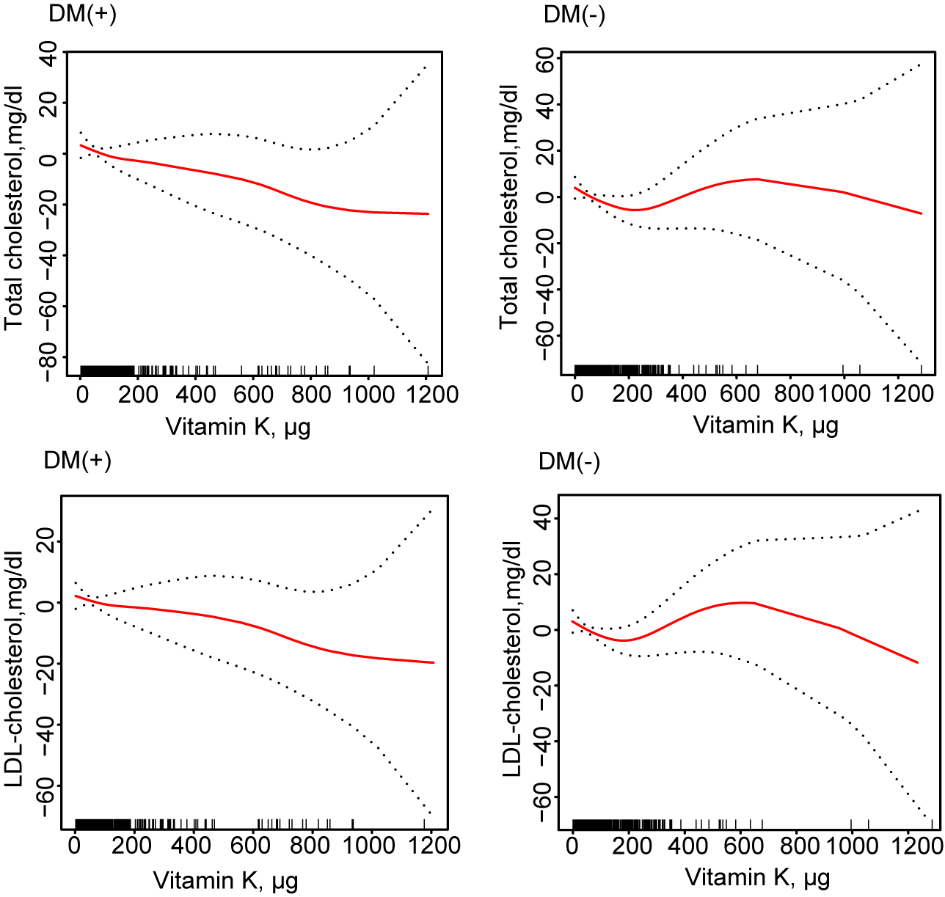


**Figure S1** Fitting curves of association of dietary vitamin K intake with TC and LDL-C among CVD populations, stratified by DM subgroups.

Age, sex, ethnicity, marital status, poverty income ratio, BMI, education level, smoking status, drinking status, hypertension, CKD, and lipid-lowering drugs were adjusted.

**Abbreviations:** HDL, high-density lipoprotein; LDL, low- density lipoprotein; BMI, body mass index; DM, diabetes mellitus; CKD, chronic kidney disease; CVD, cardiovascular disease.

**Table S2.** Threshold effect analysis of dietary vitamin K intake on serum lipid-related indicators among CVD populations without DM.

| Outcomes | ***β* (95%CI)** | *P*-value |
| --- | --- | --- |
| **Total cholesterol** |  |  |
| Inflection point | 30.2 ug |  |
| ≤30.2 ug | -0.472(-0.919, -0.025) | 0.039 |
| >30.2 ug | -0.003(-0.032, 0.026) | 0.853 |
| **LDL cholesterol** |  |  |
| Inflection point | 34 ug |  |
| ≤34 ug | -0.300 (-0.621, 0.021) | 0.067 |
| >34 ug | 0.002(-0.023, 0.027) | 0.842 |

Age, sex, ethnicity, marital status, poverty income ratio, BMI, education level, smoking status, drinking status, DM, hypertension, CKD, and lipid-lowering drugs were adjusted.

**Abbreviations:** HDL, high-density lipoprotein; LDL, low- density lipoprotein; BMI, body mass index; DM, diabetes mellitus; CKD, chronic kidney disease.

**Table S3.** Association between dietary vitamin K intake and serum lipid-related indicators according to 1297 CVD populations.

| Outcome | Model 1  β 95%CI | Model 3  β 95%CI | Model 3  β 95%CI |
| --- | --- | --- | --- |
| **Triglycerides** |  |  |  |
| Vitamin K | -0.038(-0.082,0.006) | -0.038( -0.077, 0.001) | -0.036( -0.074, 0.001) |
| Tertiles of vitamin K | |  |  |
| T1 | ref | ref | ref |
| T2 | -7.702(-17.663,2.259) | -5.436(-14.876, 4.004) | -5.007(-14.622, 4.609) |
| T3 | -14.64(-30.076,0.795) | -14.893(-28.829, -0.958) * | -14.573(-28.109, -1.036) * |
| **Total cholesterol** | |  |  |
| Vitamin K | -0.015(-0.050,0.020) | -0.004(-0.036, 0.028) | -0.005( -0.037, 0.027) |
| Tertiles of vitamin K | |  |  |
| T1 | ref | ref | ref |
| T2 | -4.08(-11.310, 3.151) | -0.398( -7.426, 6.629) | -0.915( -7.599, 5.769) |
| T3 | -11.833(-18.795, -4.871) ** | -6.329(-12.938, 0.280) | -7.326(-14.054, -0.598) * |
| **HDL cholesterol** | |  |  |
| Vitamin K | -0.002(-0.010,0.007) | 0.002(-0.004, 0.008) | 0.001(-0.005, 0.007) |
| Tertiles of vitamin K | |  |  |
| T1 | ref | ref | ref |
| T2 | 1.118(-1.129,3.365) | 1.766(-0.173, 3.705) | 1.642(-0.166, 3.450) |
| T3 | -0.431(-3.252,2.390) | 1.391(-1.148, 3.930) | 1.108(-1.323, 3.539) |
| **LDL cholesterol** | |  |  |
| Vitamin K | -0.006(-0.037,0.025) | 0.002(-0.026, 0.029) | 0.001( -0.026, 0.028) |
| Tertiles of vitamin K | |  |  |
| T1 | ref | ref | ref |
| T2 | -3.659( -9.848, 2.530) | -1.08( -7.153, 4.994) | -1.562( -7.440, 4.316) |
| T3 | -8.467(-14.225, -2.710) ** | -4.735(-10.377, 0.907) | -5.515(-11.261, 0.230) |

*: *P*-value <0.05; **: *P*-value <0.01; ***: *P*-value <0.001

Model 1 did not adjust any variables,

Model 2 adjusted age, sex, ethnicity, marital status, poverty income ratio, BMI, and education level.

Model 3 adjusted age, sex, ethnicity, marital status, poverty income ratio, BMI, education level, smoking status, drinking status, DM, hypertension, CKD, and lipid-lowering drugs.

**Abbreviations:** HDL, high-density lipoprotein; LDL, low- density lipoprotein; BMI, body mass index; DM, diabetes mellitus; CKD, chronic kidney disease; CVD, cardiovascular disease.


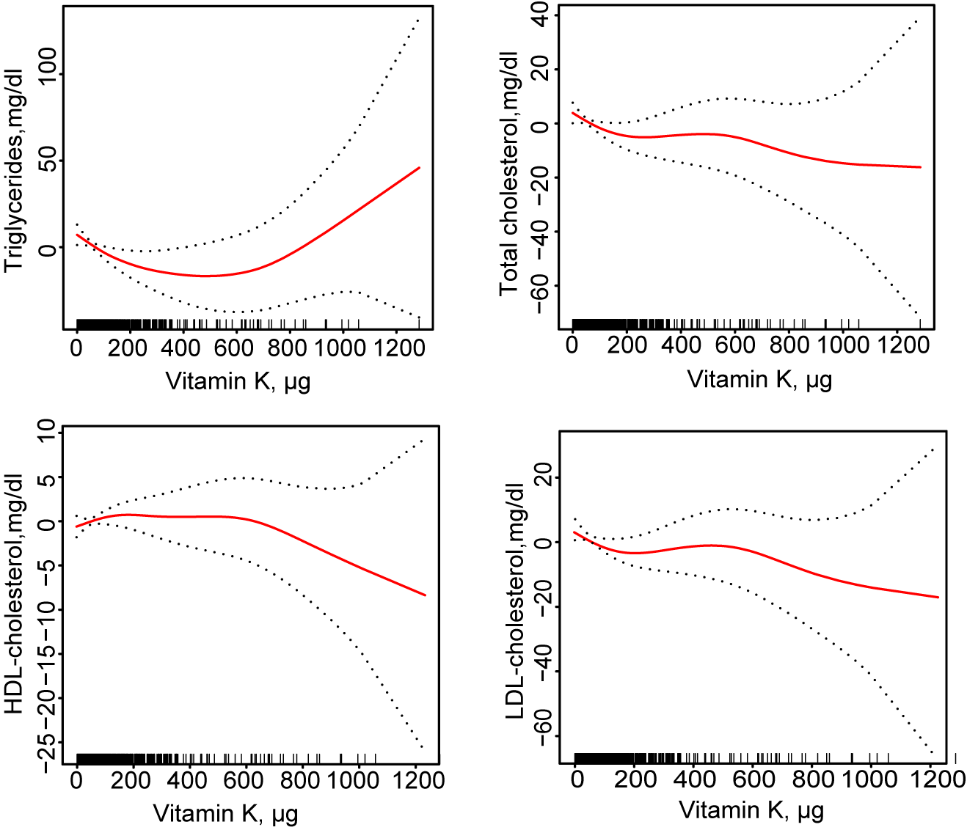


**Figure S2** Fitting curves of association between dietary vitamin K intake and serum lipid-related indicators among 1297 CVD populations.

Age, sex, ethnicity, marital status, poverty income ratio, BMI, education level, smoking status, drinking status, DM, hypertension, CKD, and lipid-lowering drugs were adjusted.

**Abbreviations:** HDL, high-density lipoprotein; LDL, low- density lipoprotein; BMI, body mass index; DM, diabetes mellitus; CKD, chronic kidney disease; CVD, cardiovascular disease.

**Table S4.** Threshold effect analysis of dietary vitamin K intake on serum lipid-related indicators among 1297 CVD populations.

| Outcomes | ***β* (95%CI)** | *P*-value |
| --- | --- | --- |
| **Triglyceride** |  |  |
| Inflection point | 237.6 ug |  |
| ≤237.6 ug | -0.109(-0.176, -0.042) | 0.002 |
| >237.6 ug | 0.016(-0.036, 0.069) | 0.537 |
| **Total cholesterol** |  |  |
| Inflection point | 96.7 ug |  |
| ≤96.7 ug | -0.103(-0.188, -0.017) | 0.018 |
| >96.7 ug | -0.006(-0.030, 0.018) | 0.609 |
| **HDL cholesterol** |  |  |
| Inflection point | 10.5 ug |  |
| ≤10.5 ug | -0.696(-1.435, 0.043) | 0.065 |
| >10.5 ug | 0.002(-0.004, 0.008) | 0.563 |
| **LDL cholesterol** |  |  |
| Inflection point | 96 ug |  |
| ≤96 ug | -0.085(-0.158, -0.011) | 0.025 |
| >96 ug | -0.002(-0.023, 0.018) | 0.821 |

Age, sex, ethnicity, marital status, poverty income ratio, BMI, education level, smoking status, drinking status, DM, hypertension, CKD, and lipid-lowering drugs were adjusted.

**Abbreviations:** HDL, high-density lipoprotein; LDL, low- density lipoprotein; BMI, body mass index; DM, diabetes mellitus; CKD, chronic kidney disease.
